# Educational material production and validity: educational instrument for home care for premature newborns

**DOI:** 10.1590/0034-7167-2021-0648

**Published:** 2023-01-30

**Authors:** Isabela Lorencini Santos, Luciana de Cássia Nunes Nascimento, Marta Pereira Coelho, Paula de Souza Silva Freitas, Adriana Nunes Moraes-Partelli

**Affiliations:** I Universidade Federal do Espírito Santo, Centro Universitário Norte do Espírito Santo. São Mateus, Espírito Santo, Brazil; II Universidade Federal do Espírito Santo, Centro de Ciências da Saúde. Vitória, Espírito Santo, Brazil

**Keywords:** Educational and Promotional Materials, Infant, Premature, Intensive Care Units, Neonatal, Health Education, Validation Study, Materiales Educativos y de Divulgación, Recién Nacido Prematuro, Unidades de Cuidado Intensivo Neonatal, Educación en Salud, Estudio de Validación, Materiais Educativos e de Divulgação, Recém-Nascido Prematuro, Unidade de Terapia Intensiva Neonatal, Educação em Saúde, Estudos de Validação

## Abstract

**Objective:**

to produce and validate a booklet, based on Jean Watson’s Theory, on home care for premature newborns, based on caregivers’ experiences.

**Method:**

a methodological study, developed in the following stages: diagnosis of knowledge needs about home care; survey of scientific content; educational material production; and validity by judges/experts.

**Results:**

the literature review resulted in 19 articles. The main themes (breastfeeding, bath care, bond building, infection prevention and support network) were listed for producing the booklet “Booklet for Premature Newborns: Demystifying Home Care”. The booklet content and appearance received the overall Content Validity Index of 0.85, considered suitable within the scientific rigor of validity.

**Final considerations:**

the booklet produced and validated is an educational material whose main role is to provide knowledge and awaken caregivers’ autonomy in providing home care to newborns.

## RESUMO

**Objetivo:**

produzir e validar cartilha, baseado na Teoria de Jean Watson, sobre os cuidados domiciliares ao recém-nascido prematuro, a partir de vivências de cuidadores.

**Método:**

estudo metodológico, desenvolvido nas etapas: diagnóstico das necessidades de conhecimento sobre os cuidados domiciliares; levantamento dos conteúdos científicos; produção de material educativo; e validação por juízas/*experts*.

**Resultados:**

a revisão da literatura resultou em 19 artigos. Elencaram-se os principais temas (amamentação, cuidados com o banho, construção de vínculo, prevenção de infecções e rede de apoio), para produção da cartilha intitulada “Cartilha de Cuidados com o Recém-Nascido Prematuro: Desmistificando o Cuidar no Domicílio”. O conteúdo e aparência da cartilha recebeu o Índice de Validade de Conteúdo Global de 0,85, considerado adequado dentro do rigor científico de validade.

**Considerações finais:**

a cartilha produzida e validada é um material educativo cujo papel principal é proporcionar conhecimento e despertar a autonomia dos cuidadores na prestação dos cuidados domiciliares ao recém-nascido.

## RESUMEN

**Objetivo:**

producir y validar una cartilla, basada en la Teoría de Jean Watson, sobre el cuidado domiciliario del recién nacido prematuro, a partir de las experiencias de los cuidadores. Método: estudio metodológico, desarrollado en las siguientes etapas: diagnóstico de las necesidades de conocimiento sobre el cuidado domiciliario; encuesta de contenido científico; producción de material educativo; y validación por jueces/expertos.

**Resultados:**

la revisión de la literatura resultó en 19 artículos. Se enumeraron los temas principales (lactancia materna, cuidado del baño, creación de vínculos, prevención de infecciones y red de apoyo) para la producción del folleto “Cuaderno para Recién Nacidos Prematuros: Desmitificando el Cuidado Domiciliario”. El contenido y apariencia del cuadernillo recibió el Índice de Validez de Contenido Global de 0,85, considerado adecuado dentro del rigor científico de la validez.

**Consideraciones finales:**

la cartilla producida y validada es un material educativo que tiene como función principal proporcionar conocimientos y despertar la autonomía de los cuidadores en la atención domiciliaria al recién nacido.

## INTRODUCTION

Infants considered premature are those born before 37 weeks of gestational age. According to the World Health Organization (WHO), in 2018, 30 million premature children were born worldwide, and in Brazil, these numbers reached about 12%. Given the magnitude of incidence and risks of prematurity, it is considered a public health concern. The WHO declared, in a report developed in 2018, that an additional investment of US$ 0.20 cents per person could save 2 in 3 newborns in low- and middle-income countries by 2030^(1)^.

In addition to being hospitalized for an extended period in the Neonatal Intensive Care Unit (NICU), the birth of a premature baby is also characterized as a stressful event, generating several feelings in caregivers, such as fear, anxiety, unpreparedness, in addition to of several doubts about the prognosis and the care that will be exercised at home after hospital discharge^(2-3)^.

Given this scenario, hospital discharge must be based on planning, with the main purpose of training and informing caregivers about continuity of care at home. Thus, health professionals must provide health education where the guidelines are made available, in a clear, concise way, with accessible language and clear understanding for family members^(4)^. Thus, educational health technologies can be an auxiliary tool to complement the care information provided by health professionals.

To solidify the scientificity of an educational technology in health, it can be based on a nursing theory. This action promotes the connection between theory and practice so that the ideas and experiences of professionals are scientifically attested, becoming a timely source of knowledge to be used^(5)^. In this context, the Theory of Transpersonal Caring is described as an interactionist theory, as its execution takes place through direct and indirect communication between patient and nursing team. This mutual correlation in care is an experience that requires dialogue between the subjects involved in the health-disease process, contributing to a care practice that takes into account the satisfaction of human needs, health promotion and individual and family growth, as well as in the understanding of the environment as favoring personal development and integrated with biophysical and human behavioral knowledge^(6)^.

During hospitalization, the nursing team is in constant contact with caregivers, who are usually the mothers, providing information about the care provided to premature newborns (PTNB), and, in a timely manner, provides guidance with a focus on hospital discharge. However, there is evidence that the preparation of caregivers for hospital discharge is weakened. Several mothers and caregivers, even after experiencing the preparation for hospital discharge, showed insecurity in fully taking care of their babies at home^(7)^.

At the time of hospital discharge, care with the hygiene and nutrition of newborns is often the subject of doubts and insecurity by families. This care was analyzed by Santos, Góes, Ledo et al.^(8)^ in a study that raised the learning demands of puerperal women and their families about postnatal care for newborns. The authors observed that, mistakenly, families performed some practices linked to cultural heritage, contrary to scientific recommendations and may endanger newborns’ health.

In this context, the use of educational technologies in health has been gaining considerable space in terms of health education for the population, due to its potential to encourage individual and collective empowerment through information and knowledge relevant to the topic. They can be beneficial to caregivers, with instructions that can improve the strengthening of affective bonding and reduce insecurity in premature baby care^(8-10)^.

And, for a better effectiveness of health technology and achievement of the proposed objectives in its structuring, it is necessary to go through the validity stage. Educational technology validity, such as booklets, manuals, videos, games and others, has been commonly used for a better development and improvement of the health education offered, as well as for the improvement of teaching-learning strategies. Thus, it is possible to share knowledge in a simpler way, clarify doubts and promote the participation of all those involved, making it also an instrument of greater reliability regarding the content made available^(11)^.

## OBJECTIVE

To produce and validate a booklet, based on Jean Watson’s theory, on home care for PTNB, based on caregivers’ experiences.

## METHODS

### Ethical aspects

The research was approved by the Research Ethics Committee of the *Centro Universitário do Norte do Espírito Santo*, of the *Universidade Federal do Espírito Santo* (UFES), through the Brazil Platform, in accordance with Resolution 466/2012^(12)^. All participants signed the Informed Consent Form (ICF). To ensure participant anonymity, they were identified by the letter “J”, followed by the number of the completed instrument (J1, J2, etc.).

### Study design, period, and location

This is a methodological study that integrates a quantitative and qualitative approach, focusing on educational material production and validity^(13)^, carried out between May 2019 and December 2020, in São Mateus, Espírito Santo, with four stages: 1) Diagnosis of knowledge needs about home care reported by caregivers, evidenced by literature review; 2) Survey of scientific content on home care for PTNB through bibliographic research; 3) Production of educational material on home care for PTNB, based on Jean Watson’s Theory of Transpersonal Caring; 4) Validity of educational material by judges/experts.

### Study protocol

In the first stage, a literature review was carried out^(14)^ to answer the guiding question: what are the main doubts regarding home care for PTNB after hospital discharge? The articles were extracted from the following databases: Medical Literature Analysis and Retrieval System Online (MEDLINE); Latin American and Caribbean Center on Health Sciences Information (LILACS); and PubMed. A library, the Scientific Electronic Library Online (SciELO), was also used. We used the Descriptors in Health Sciences (DeCS) (“Discharge planning”; “Premature newborn”; “Neonatal Intensive Care Unit”; “Mother-child bond”) in the databases, associated through the Boolean operator AND.

We included works published between 2008 and 2018, in Portuguese, English and Spanish, available for full reading. The main themes/problem situation were obtained as results: difficulty in continuing breastfeeding; bath care; maintenance of body temperature; relevance of building the mother-child bond; importance of family support network; and infection prevention.

In the second stage, we carried out bibliographic research on each theme/problem situation observed in the literature, from the perspective of mothers and caregivers, to incorporate it into the educational material to be produced and validated.

To carry out the bibliographic research, guiding questions were elaborated regarding the selected themes: what are the imminent difficulties in breastfeeding PTNB at home? What are the contributions of mother-child bond construction to PTNB? What is the imminent newborn care at bath time? How important are infection prevention measures for PTNB and their repercussions on health maintenance? What are the impacts of support networks in helping PTNB parents after hospital discharge?

Considering the guiding questions, we defined the descriptors within each problem situation, namely: Breastfeeding; Premature Newborn; Health Education; Discharge Planning; Bathing; Premature Newborn; Health education; Discharge Planning; Mother-Child Interaction; Premature Newborn; Health education; Discharge Planning; Primary Prevention; Premature Newborn; Health education; Family; Premature Newborn; Support Network; Discharge Planning.

In order to structure the current scenario of literature that addresses the relevance of each proposed theme, a manual search was carried out in databases of the Ministry of Health and in the CAPES theses and dissertations database. The databases used were LILACS and SciELO, with access via the CAPES Portal, from October to December 2019. The investigation in the literature was carried out by combining the descriptors mentioned above, using the Boolean operator AND.

Studies that proved the merit of each topic addressed and its direct influence on the maintenance of PTNB’s health at home after hospital discharge were included in the bibliographic research. These could be presented in Portuguese, Spanish and English, published between 2015 and 2019, and which were available for reading in full.

Then, we moved on to the third stage, where each issue/problem situation on home care to PTNB was analyzed in the light of Jean Watson’s Theory of Transpersonal Caring. This foundation in the construction of educational tools is necessary to support both theoretical and practical knowledge in a nursing theory^(5)^. Jean Watson’s Theory of Transpersonal Caring advocates a conscious intervention in care, emphasizing healing and integrity; unites conventional science (technical care) and modern nursing practices (educational health technologies), so that they complement each other; generates a greater understanding regarding the raising of any questions about the meaning of caring; and emphasizes the preservation of health and seeks ways to protect, enhance and promote a person’s dignity, humanity, integrity and inner harmony^(15-16)^.

For the booklet structuring and construction, a provisional storyboard was prepared, according to the format described by Campos^(17)^. Thus, the provisional storyboard was assembled in a simple, contemporary way, with few rules. It was written in Courier New, and the narrator’s lines, in Times New Roman, in Word for Windows. The provisional booklet “TITLE” was centered on the page and in capital letters. The numbering was performed frame by frame at the top, on the left, for example: Chart 1. Each frame was composed by caricature presentation of characters, people and places, and descriptions of characters or places should not exceed a few lines. The dialogue was written inside the frame, so that the character’s speech was inside a speech bubble surrounded by the descriptions. In one or several lines, dialogues are always in space 1. The provisional storyboard was delivered to a professional working in the area of graphic design, who illustrated and diagrammed the final version of the booklet.

**Chart 1:** Application of Jean Watson’s Theory of Transpersonal Caring in home care inherent to premature newborns, São Mateus, Espírito Santo, Brazil, 2020

Elements of Watson’s Theory	Care addressed in educational material
Practice kindness and equanimity, including for yourself.	Mother-child bond construction; Importance of a support network.
Being present and valuing the care system’s belief system.	Breastfeeding; Mother-child bond construction; Bath care; Importance of a support network; Infection prevention.
Cultivate one’s own spiritual practices, deepening individual knowledge.	Mother-child bond construction; Importance of a support network.
Maintain authentic caring through a helping-trusting relationship.	Breastfeeding. Mother-child bond construction; Bath care; Infection prevention.
Maintain authentic caring through a helping-trusting relationship.	Mother-child bond construction; Importance of a support network.
Use knowledge and intuition creatively in problem solving.	Breastfeeding; Bath care; Infection prevention.
Truly engage in the teaching-learning experience.	Breastfeeding; Mother-child bond construction; Bath care; Importance of a support network; Infection prevention.
Provide an environment of physical, emotional and spiritual restoration.	Breastfeeding; Mother-child bond construction; Bath care; Importance of a support network; Infection prevention.
Promote alignment of body, mind and spirit in order to meet individuals’ needs.	Breastfeeding; Mother-child bond construction; Bath care; Infection prevention.
Consider the spiritual and life and death aspects.	Mother-child bond construction; Importance of a support network.

*Source: adapted from the table “Phenomenological existential dimension: the sacredness of being”^(21)^.*

In the fourth step, the process of validating the booklet content and appearance took place with the following steps: selection of judges/experts; data collection and data analysis.

### Participants

Judges/experts were selected through the Lattes Curriculum of the Brazilian National Council for Scientific and Technological Development (CNPq - *Conselho Nacional de Desenvolvimento Científico e Tecnológico*) portal, using the subject search with the keywords: Discharge Planning; Premature Newborn; Neonatal Intensive Care Unit; Mother-Child Bond; Home Care; Validity Studies and Health Education, in the simple search option and use of filters to refine the criteria The judges/experts who achieved a score equal to or greater than five points were included according to the criteria of expertise^(18)^: titration; scientific production on the topic under discussion; and time of action with the theme under discussion. Professionals who did not reach the minimum score established were excluded.

### Data collection

The specialists’ work consisted of a critical reading of the information contained in the booklet and the completion of an adapted assessment instrument, the Suitability Assessment of Materials, which is characterized as a semi-structured questionnaire with a Likert-type scale to assess the items. comprises scores from one to five, with the description of each item being: (1) Do not agree; (2) Partially agree; (3) Neither agree nor disagree; (4) Agree more than disagree; (5) Totally agree. Each statement corresponds to an assessment item, distributed in six assessment domains (content, writing, illustrations, presentation, motivation and cultural suitability). There are also spaces for general suggestions and comments^(19)^.

Contact with study participants was carried out by invitation letter, sent via e-mail with clarifications about the research, its objectives, the ICF, as well as the guidelines for completing the instrument by Google Forms, whose estimated time was approximately 20 minutes.

Initially, 35 judges/experts were selected, however 12 responded and agreed to participate in the validity process. Thus, a period of 15 days was established for the return of the analyzed material, but there was a need to increase the period, and data collection was completed with 25 days.

Finally, the information described by judges/experts was consolidated, proceeding with the considerations and suggested reformulations of the booklet, according to suggested recommendations.

### Data analysis

Data were entered into the Microsoft Office Excel 7.0 program, organized in tables and charts and analyzed according to the Content Validity Index (CVI), calculating based on three mathematical equations: S-CVI/Ave (average of content validity indices for all scale indices); S-CVI/UA (proportion of scale items that reach a score of 4/agree and 5/totally agree, by all judges/experts); and I-CVI (content validity of individual items)^(20)^. It is worth noting that the CVI varies from -1 to 1, and the item whose agreement between judges/experts is equal to or greater than 0.80 is considered valid^(19)^.

The items that obtained a mean lower than the CVI established in the study were modified through the suggestions given by judges/experts. The suggestions of each professional for improving the booklet were analyzed, following them.

## RESULTS

### Educational booklet production

The integrative literature review made up the final quantified sample, nineteen publications, eleven from SciELO and eight from LILACS. It was observed that 52.63% assesses the experience of maternity in relation to PTNB birth and the feelings in relation to hospital discharge; 31.57% assessed the promotion of imminent health education to PTNB caregivers in the NICU, carried out by the health team; and 15.78% assessed the beliefs and practices of PTNB caregivers at home. As for the implications for practice, 100% of studies showed positive implications related to caregivers’ feelings and questions regarding PTNB home care, in addition to the need to improve health education to prepare caregivers for hospital discharge. Thus, it was possible to select the main themes/situations problems addressed in the publications.

As a structure for the educational material construction, the Theory of Transpersonal Caring, idealized by Jean Watson, was adopted. Chart 1 shows the application of Jean Watson’s theory ten elements to the five most frequent themes, raised by the integrative literature review, and which were addressed in the educational material.

Following the steps in the educational material production, the booklet was entitled “Care booklet for Premature Newborns: Demystifying Home Care”, composed of cover and 41 pages, with standard formatting size of 21 cm high by 15 cm wide.

For the dialogic educational material production, the first step was to define who would dialogue with caregivers at home. In this way, the biographical description, the name and the caricature of a character who would be the mediator of this dialogue were defined, electing “nurse Catarina”. It is worth mentioning that this character is fictitious and was inspired by the researchers’ practical experience in carrying out health education with children and adolescents.

Then, illustrations, textual content and language used were defined. The storyboard was organized and structured with tables, listed in the sequence of the facts, containing narrated texts in the upper portion, balloons with nurses’ dialogue and images in the center of the table. To help the illustrator with the researchers’ ideas, a description of the scenes was carried out at the end of the frame, and the images were taken from the free domain, available on electronic search pages.

Scientific information was incorporated into the educational material in the format “Curiosities” and “Did you know?”. Curiosities were inserted as a short and direct text, right after the character’s exposition of the problem situation. In this way, the character dialogues with the reading public, encouraging them to reflect on each theme within their reality.

**Figure 1:**
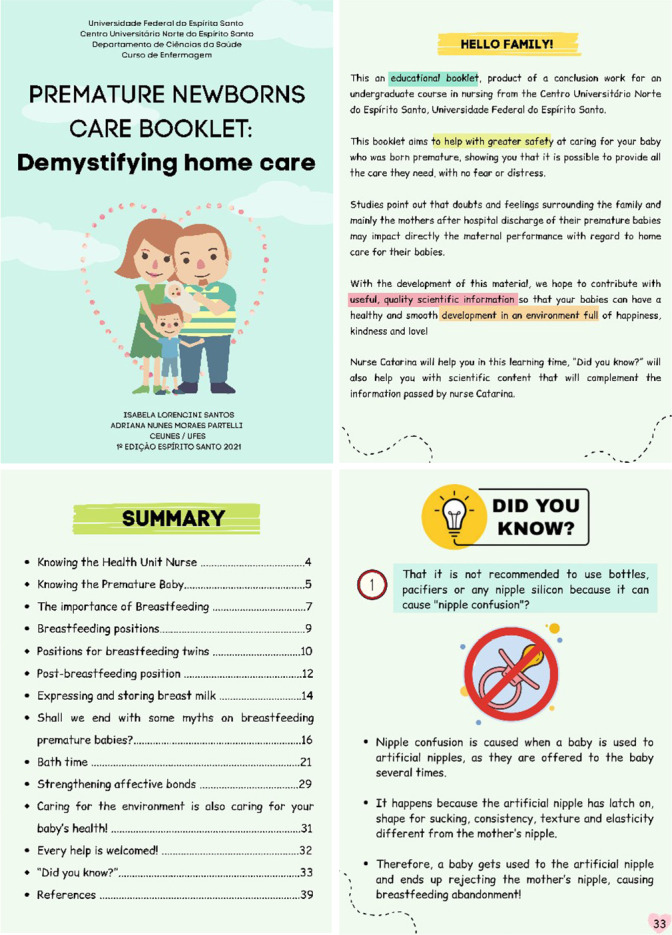
Cover, presentation, summary and “Did you know?” of the educational booklet produced and validated in this study. São Mateus, Espírito Santo, Brazil, 2021

Already the information of “Did you know?” they were inserted in a separate text, at the end of the booklet, as complementary, broader content and referring to the context of the information described by the booklet character. The following topics were addressed: nipple confusion; offering milk in the glass; care when offering milk in the glass; consequences of using artificial nipples; relationship between twin pregnancy and preterm birth; and PTNB warning signs (Figure 1).

### Validity by judges/experts

A total of 12 judges/experts participated in the validity process, of which 100% were female; 50.00% were between 30 and 40 years old; 33.33% were between 41 and 50 years old; and 16.16% were between 51 and 60 years old. All judges/experts had a degree in nursing, worked in teaching; 75.00% were between 11 and 25 years old; and 25.00% had 2 to 10 years of specific activity in the area of this study.

The minimum score required was 5 points. The score achieved by health judges/experts in the classification system obtained an average of 16.16, demonstrating considerable expertise in the broad areas of this study.

As for the educational technology validity process, the following CVI results were verified for the categories: content: 0.91; writing: 0.84; illustrations: 0.87; presentation: 0.62; motivation: 0.93; and cultural suitability: 0.91. Sub-item 4.2 (Are the font size and type appropriate?) of presentation received a score of 0.6, with the type and font size being duly changed. As the educational material reached an overall CVI of 0.85, it was considered validated (Table 1).

**Table 1:** Content Validity Index scores as assessed by health judges/experts, São Mateus, Espírito Santo, Brazil, 2021

Assessment items	n (N=12)	CVI^*^
1. Content		0.91
1.1 Does the booklet help to resolve doubts of parents and/or caregivers in relation to PTNB^**^ home care?	11	0.91
1.2 Is the booklet content directly related to PTNB home care?	12	1.0
1.3 Is content suitable to answer questions from parents and/or caregivers regarding PTNB home care?	10	0.83
1.4 Does content address relevant topics about PTNB home care?	11	0.91
2. Writing		0.84
2.1 Is the information in the booklet easy to understand?	10	0.83
2.2 Is reading clear and concise?	10	0.83
2.3 Are the words used common in everyday life?	10	0.83
2.4 Is there agreement between information?	10	0.83
2.5 Do topics facilitate reading comprehension?	11	0.91
3. Illustrations		0.87
3.1 Do images/figures contribute to understanding the information?	10	0.83
3.2 Are images/figures suitable for the type of material?	10	0.83
3.3 Are images/figures important?	12	1.0
3.4 Do images/figures contain clear information?	10	0.83
4. Presentation		0.62
4.1 Is the overall appearance (cover and all other pages) attractive and appropriate?	09	0.75
4.2 Are the font size and type suitable?	06	0.50
5. Motivation		0.93
5.1 Is the material motivating?	11	0.91
5.2 Are the guidelines clear and do they cite examples?	11	0.91
5.3 Does the booklet encourage behavior change?	11	0.91
5.4 Does the booklet make it possible to acquire new knowledge?	12	1.0
6. Cultural suitability		0.91
6.1 Is the booklet a material appropriate to the reality of life of parents and/or caregivers?	11	0.91
6.2 Does the booklet address information that is often present in the daily life of parents and/or caregivers?	11	0.91
OVERALL CVI	0.85	

* CVI=Content Validity Index;

** PTNB=premature newborns.

The relevance of comments and suggestions was highlighted, as a result of the booklet analysis by judges/experts with expertise in the investigated subject, which enhanced the educational material with their considerations, in order to achieve the objectives outlined.

The main positive points listed refer to the relevance of the theme choice (J3, J5, J6); up-to-date scientific information, information in a simple way, covering people with different levels of knowledge (J9); incorporation of images with different skin tones, highlighting racial diversity and the importance of representation (J12); and for being an excellent tool to help PTNB mothers, especially with regard to promoting the safety of caregivers in the execution of home care (J9 and J12).

Although only the presentation item did not reach the target value, judges/experts presented some relevant changes to improve the educational material as a whole, which were accepted.

Specifically on the booklet content, judges/experts assessed: replace the way of measuring the water temperature for the bath for the wrist region (J4); remove the sunbathing topics, as it is no longer recommended by the WHO; replace information about anterior and posterior milk with the link between the mother’s relaxation state added to oxytocin peaks and the breast milk ejection reflex (J7); replacement of brown fat content with information about heat loss; and maintenance of PTNB temperature (J8 and J12).

With regard to writing, judges/experts judged: to exchange some technical terms for simpler terms to facilitate the understanding of the target audience (J2 and J8); replace single spacing with 1.5 or double spacing (J3); write NICU in full for better understanding the term (J6); use verbs in the infinitive and standardize the punctuation at the end of sentences (J8), in addition to carrying out a textual review (J4, J10, J11 and J12).

As for illustrations, judges/experts commented on the need to exchange some illustrations for smaller and more delicate ones (J5, J6 and J11) and add the reference of the images (J11).

Regarding the booklet presentation, judges/experts judged the need to change the font and use capital letters only for titles and important topics (J1, J2, J3, J4, J5 and J12).

And as for the topic of cultural suitability, only one judge/expert commented on the incorporation of information that the educational material does not address all the answers to the questions that will arise on a daily basis, therefore, they included information about the importance of attending childcare consultations (J12).

It is worth emphasizing that, in the comments and/or suggestions, limitations in spelling and agreement were pointed out; thus, the material underwent textual review by a qualified professional.

## DISCUSSION

In the present study, an educational material was produced and validated in a booklet format, based on Jean Watson’s Theory of Transpersonal Caring, on PTNB home care, based on caregivers’ experiences.

Educational technologies are commonly used in the field of health, addressing a wide variety of themes for different target audiences. Within this scope, technologies are available such as: manuals for the execution of a safe surgery^(22)^; educational games that deal with depression in adolescence^(23)^; serial album on acquired syphilis^(24)^; educational video for guidance of parents of children in clean intermittent catheterization^(11)^; Illustrative guide to health education for people who have experienced a stroke^(25)^; almanac and games that address the issue of alcohol in adolescence residing in a *Quilombola* community^(26-28)^; educational video on newborn care in the knowledge of pregnant women, postpartum women and family members, among others^(30)^.

In order to develop quality educational material based on the reality of the experience of individuals, groups and communities, studies show that one of the ways to produce it is by carrying out a survey of content/problem situations to be addressed through a literature review^(24,29,31)^. In the present study, a literature review directed to five themes, which were addressed in the booklet, meeting the target audience’s real needs. In order for the foundation of listed themes to occur reliably and based on scientific knowledge, a bibliographic research was carried out, to identify the current stage of knowledge of themes that would be present in the booklet^(24)^.

Thus, the booklet was structured on Jean Watson’s Theory of Transpersonal Caring, which advocates a conscious intervention in care, emphasizing healing and integrity. It unites conventional science and modern nursing practices so that they are complementary to each other. Thus, the booklet was produced with the foundations of theory, that is, the booklet brings scientific content and aspects related to human needs as a whole (holistic), biological, community, social and spiritual, aiming to encourage caregivers’ autonomy, self-control and self-knowledge in carrying out PTNB home care^(5,32)^. It is noteworthy that the booklet presents itself as a consultation option for PTNB caregivers in home care, as it contains scientific information in a simple way, and can be consulted at any time.

Another aspect of Watson’s Theory used in the booklet was the nurse-patient interaction, establishing dialogue between people. To this end, a fictitious character (nurse Catarina) was created, in order to establish the construction of a process of interaction between health professionals (nursing team) and social subjects (caregivers), in a horizontal relationship. The use of fictional characters is also observed in other studies^(32-35)^. In this context, the use of fictional characters promotes the construction of health knowledge in a shared and simplified way, favoring the scope and the incorporation of scientific knowledge. Moreover, dialogue promotes empowerment, a fact that is closely related to overcoming situations of insecurity and, consequently, generates the transformation of their realities^(36)^.

In addition to the dialogue established by the nurse, the various illustrations used in the booklet stand out. We sought to choose illustrations focused on the reality of the social environment where the home environment was contemplated and images related to newborns’ daily care needs, such as items used in the bath, clothes, among others. Studies show that booklets made with text accompanied by illustrations attract the reader arouses interest in reading and assists in understanding the text^(37)^.

A differential of the booklet produced in relation to the format of other health booklets available stands out, which is the presentation of complementary informative and scientific content in the format of “Curiosities” and “Did you know?”, highlighting the uniqueness of the work produced and validated. The use of “Curiosities” and “Did you know?” to address the scientific content is carried out in other educational materials such as almanacs^(26-27)^.

As for validity, the booklet was considered suitable by judges/experts, with minor corrections, which were fully accepted and changed. The answers of judges/experts were analyzed, quantitatively, in search of recommended rigor in the validity process, checking the equivalence between the answers and the fulfillment of the objectives proposed in the booklet, as recommended in the literature^(38)^.

Validity by judges/experts reached satisfactory rates, reaching an overall CVI of 0.85. With an index slightly lower than this, recent research, conducted in Fortaleza, Brazil, in which educational technology was validated for the breastfeeding room, obtained overall CVI of 0.81^(39)^. In addition to this study, another study developed and validated an educational booklet for postpartum health and well-being, with an overall CVI of 0.80^(40)^.

Thus, the validity of booklet-type educational materials has shown satisfactory results in national and international studies. Therefore, it was noted the importance that the guidance instruments promote in the target audience’s reality, contributing directly to the culture of safety and autonomy both in self-care and in caring for the other^(41-42)^.

### Study limitations

The study had some limitations, such as non-validity with the target audience, a fact that occurred in the face of the moment of social isolation caused by the COVID-19 pandemic, which made face-to-face contact with possible caregivers of premature babies impossible. It is intended to continue the research, carrying out the validity to verify the understanding and effectiveness of the content contained in the booklet in the daily practice of these caregivers.

### Contributions to health

The main contribution of this study is due to its social relevance, as the construction and validity of an educational health technology in the form of a booklet will help health professionals with the information provided in the preparation for hospital discharge, guiding caregivers of babies who were hospitalized in the NICU regarding the execution of home care and promoting greater knowledge, safety and empowerment of families in providing care for their baby.

## FINAL CONSIDERATIONS

This study described the process of construction and validity of an educational technology in health, a booklet, to assist caregivers in home care of premature babies, scientifically translated. The entire methodology used proved to be able to assist in the educational booklet elaboration in a didactic, dynamic, easy to understand, attractive, dialogic and comprehensive way, which can contribute to the knowledge of people who provide care and the development of other educational technologies both in this theme and in any other that involves the need to promote health care.

It is noteworthy that the research applied a nursing theory that supported the production of an innovative health educational technology on home care for the newborn, generating new knowledge and contributing to the advancement of nursing as a science of care.

## SUPPLEMENTARY MATERIAL

It should be noted that the booklet is available and accessible free of charge in e-book format through the link http://repositorio.ufes.br/handle/10/11666

0034-7167-reben-76-01-e20210648-sup01
