# Supplementary material for: Educational material production and validity: educational instrument for home care for premature newborns
Source: Rev Bras Enferm. 2023 Jan 30;76(1):e20210648. doi: 10.1590/0034-7167-2021-0648 (PMC9885364; doi:10.1590/0034-7167-2021-0648)
Supplement: 0034-7167-reben-76-01-e20210648-sup01 [file 0034-7167-reben-76-01-e20210648-sup01.pdf]

Universidade Federal do Espírito Santo
Centro Universitário Norte do Espírito Santo
Departamento de Ciências da Saúde
Curso de Enfermagem

CARTILHA DE CUIDADOS COM O RECÉM-NASCIDO PREMATURO: **desmistificando o cuidar no domicílio**

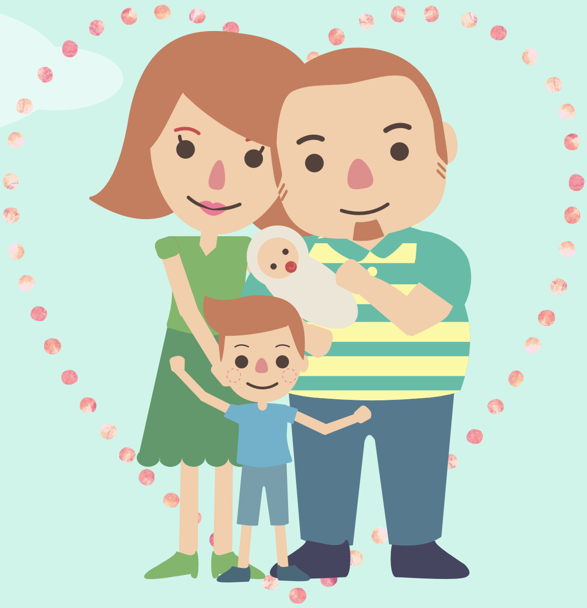

ISABELA LORENCINI SANTOS
ADRIANA NUNES MORAES PARTELLI
CEUNES / UFES
1º EDIÇÃO ESPÍRITO SANTO 2021

PRODUÇÃO

Universidade Federal do Espírito Santos
Centro Universitário Norte do Espírito Santo
Departamento de Ciências da Saúde
Núcleo de Pesquisa em Saúde
Rodovia BR 101 Norte, Km 60, Bairro Litorâneo, São Mateus - ES -CEP: 29932-540
Email: isabelalorencinii@gmail.com e adrianamoraes@hotmail.com

ORGANIZADORES

Isabela Lorencini Santos
Adriana Nunes Moraes Partelli

PROJETO GRÁFICO E ILUSTRAÇÕES

Designer: TXDDY
Ilustrador: TXDDY

REVISÃO DE TEXTO

Vanildes Rsoaine Alvarenga

Dados Internacionais de Catalogação-na-publicação (CIP)
(Biblioteca Central da Universidade Federal do Espírito Santo, ES, Brasil)

C327 Cartilha de cuidados com o recém-nascido prematuro : desmistificando o cuidar no domicílio / organizadores, Isabela Lorencini Santos, Adriana Nunes Moraes Partelli ; ilustrações, TXDDY. - São Mateus, ES : Universidade Federal do Espírito Santo, Centro Universitário Norte do Espírito Santo, 2021.
43 p. : il. ; 23 cm

Inclui bibliografia.
ISBN: 978-65-00-17168-6

1. Cartilhas. 2. Recém-nascidos. 3. Prematuros. 4. Tratamento intensivo neonatal. 5. Promoção da saúde. I. Santos, Isabela Lorencini, 1997-. II. Partelli, Adriana Nunes Moraes, 1974-.

CDU: 616-053.31

Elaborado por Michele Rodrigues – CRB-6 630

OLÁ FAMILIARES!

Essa é uma cartilha educativa, produto de um trabalho de conclusão do curso de graduação em Enfermagem do Centro Universitário Norte do Espírito Santo, Universidade Federal do Espírito Santo.

Essa cartilha tem como objetivo te ajudar a ter mais segurança na hora de cuidar do seu bebê que nasceu prematuro, te mostrando que é possível fazer todos os cuidados que ele precisa, sem medo ou angústias.

Os estudos apontam que dúvidas e sentimentos que permeiam a família e principalmente a mãe após a alta hospitalar do bebê prematuro, podem impactar diretamente no desempenho do papel materno no que diz respeito a execução do cuidado à esse bebê no domicílio.

Com a elaboração desse material, esperamos contribuir com informações úteis, científicas e de qualidade para que o seu bebê possa ter um desenvolvimento pleno e saudável, em um ambiente repleto de alegria, carinho e amor!

A Enfermeira Catarina te ajudará nesse tempo de aprendizado e o "Você sabia ?!" também te auxiliará com conteúdos científicos complementares às informações passadas pela Enfermeira Catarina.

SUMÁRIO

- Conhecendo a Enfermeira da Unidade de Saúde...4
- Conhecendo o bebê prematuro......................................5
- A importância da Amamentação....................................7
- Posições para amamentar...9
- Posições para amamentar gêmeos................................10
- Posição pós amamentação..12
- Retirando e armazenando o leite materno..................14
- Vamos acabar com alguns mitos em relação à amamentação do prematuro ?!......................................16
- Hora do banho...21
- Estreitando laços afetivos...29
- Cuidar do ambiente também é cuidar da saúde do bebê!...31
- Todo apoio é bem vindo!..32
- "Você sabia ?!" 33
- Referências...39

CATARINA É UMA ENFERMEIRA QUE TRABALHA NO "POSTINHO" DO BAIRRO HÁ CERCA DE 10 ANOS!

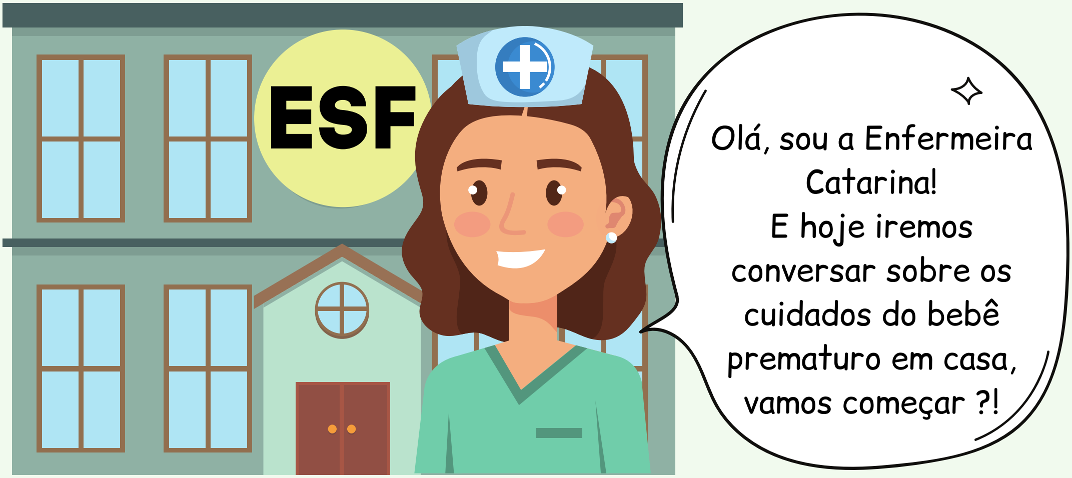

- Catarina vai realizar uma visita domiciliar após 5 dias da chegada de um bebê prematuro em casa, que ficou internado na Unidade de Terapia Intensiva Neonatal (UTIN) por 20 dias.

- O objetivo dessa visita é orientar os pais quanto aos cuidados básicos com esse bebê no domicílio, diminuindo os medos e fazendo com que os pais tenham mais autonomia ao cuidar do seu bebê, e para que dessa forma ele cresça cada vez mais saudável!

CONHECENDO O BEBÊ PREMATURO

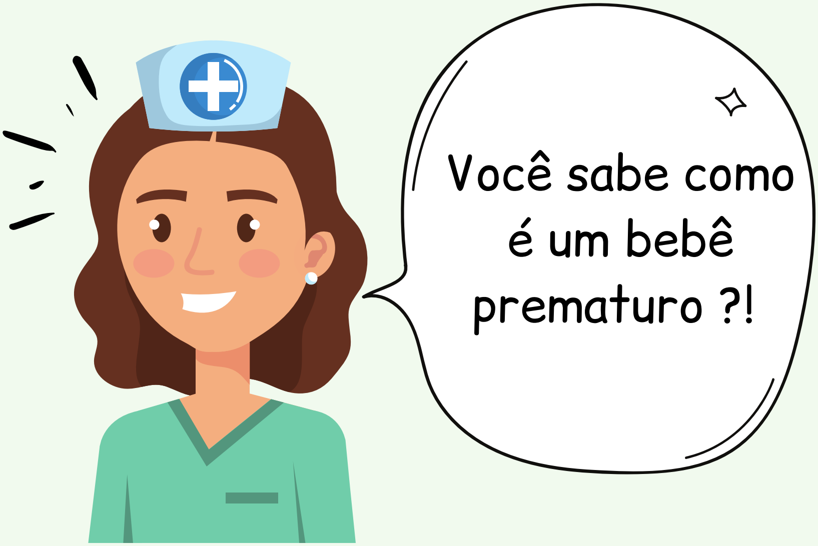

- O bebê prematuro é um bebê que nasceu depois da 20° semana e antes de completar 37 semanas de gestação, e por isso, ele apresenta alguns sistemas do corpo ainda imaturos, que precisam de cuidados especiais para que ele possa crescer e se desenvolver da melhor maneira possível!

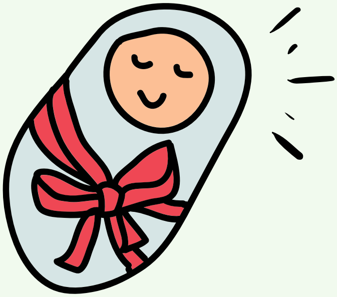

- Devido a essa imaturidade, ele pode necessitar de alguns tratamentos específicos ainda no hospital, como por exemplo:

- Ficar internado na UTIN por alguns dias;
- Utilizar o oxigênio para respirar melhor;
- Utilizar sonda para sua alimentação;
- Ficar na incubadora ou berço aquecido para manter a temperatura do corpinho;
- E algumas medidas de prevenção para evitar infecções.

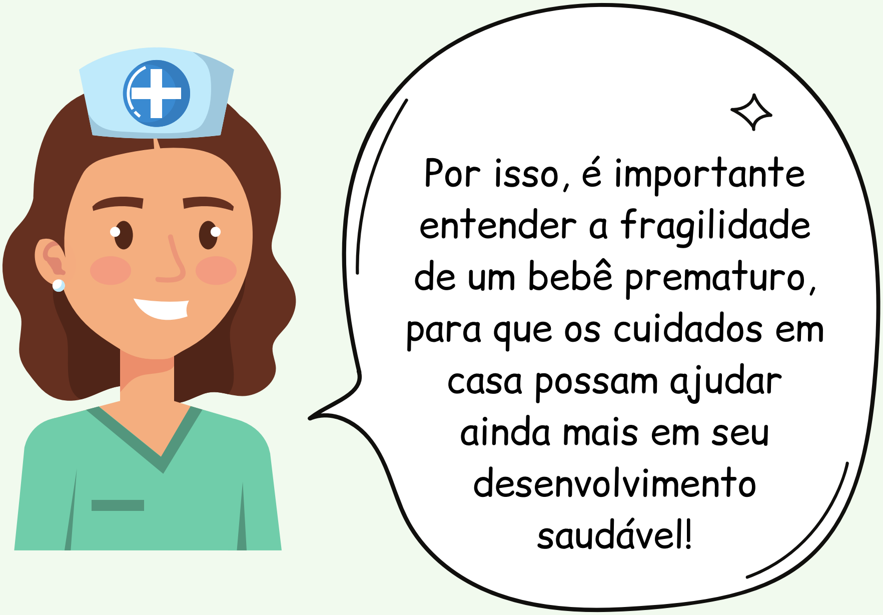

A IMPORTÂNCIA DA AMAMENTAÇÃO!

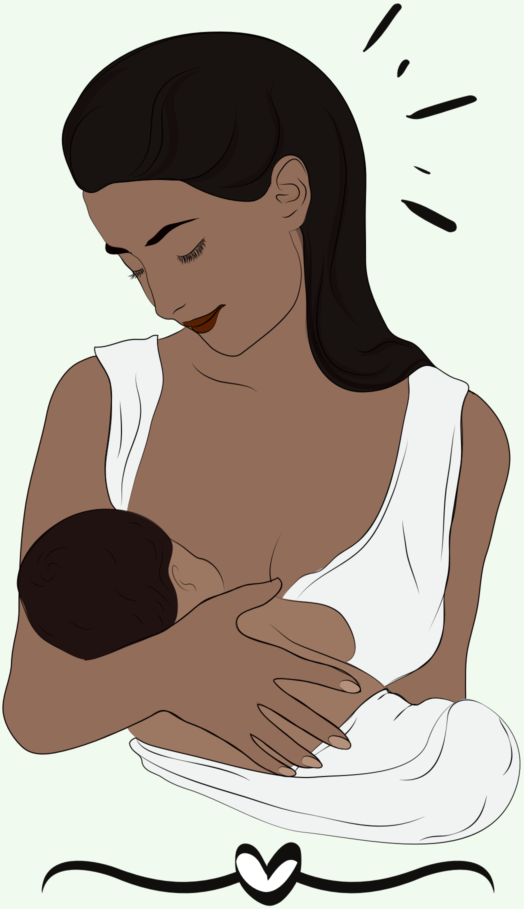

"Amamentar é um ato de amor, carinho, resistência, insistência e paciência!"

(Autor desconhecido)

Nesse momento aprenderemos como deve ser a pega correta, posições para amamentar, como fazer a retirada manual e o armazenamento do leite materno!

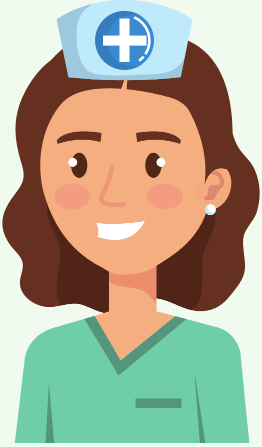

Grande parte da aréola
na boca do bebê e
não apenas o mamilo

Bochecha cheia quando suga o leite

Lábios virados para fora

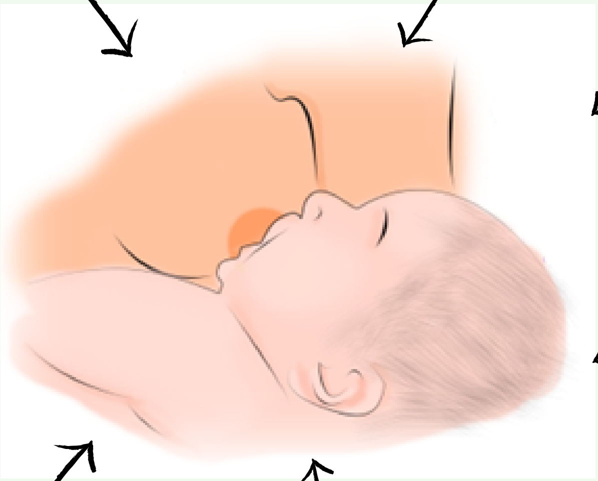

Nariz não encosta no peito e respira livremente

Boca aberta como "boquinha de peixe"

Barriga e troncos do bebê voltados para a mãe

Queixo encostado no peito

CURIOSIDADE

- Uma forma muito legal de ajudar o bebê prematuro a pegar o peito é utilizar o reflexo da procura: basta encostar o peito do ladinho da boca do bebê e esfregar com muito carinho, logo o bebê sentirá o cheiro do leite e abrirá a boca para procurar o mamilo!

POSIÇÕES PARA AMAMENTAR

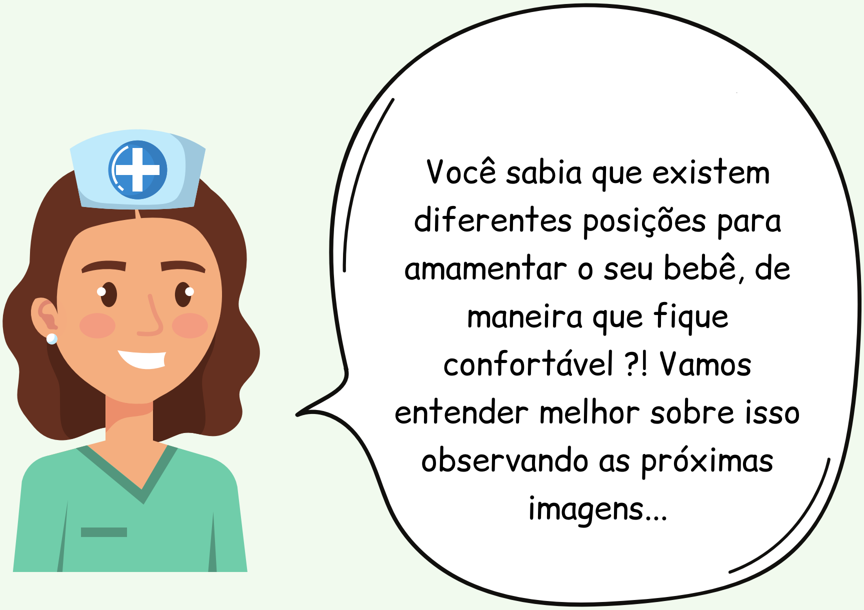

1

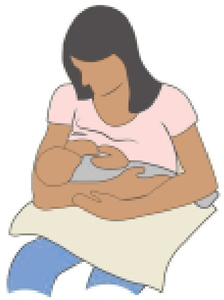

2

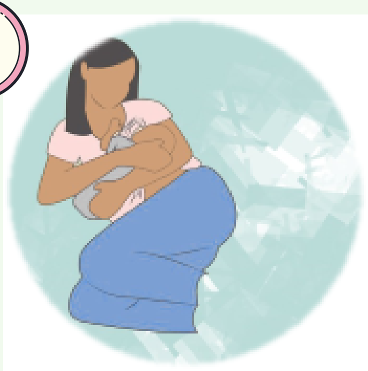

3

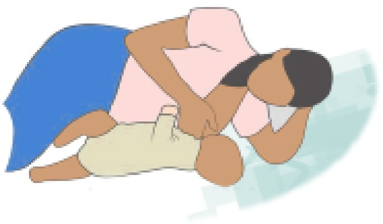

4

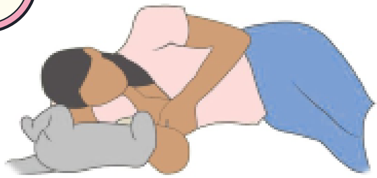

5

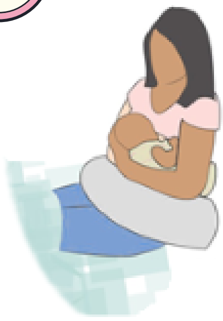

6

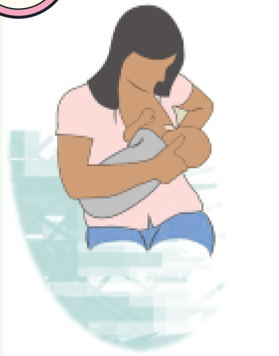

7

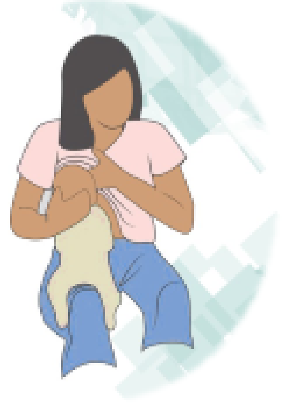

8

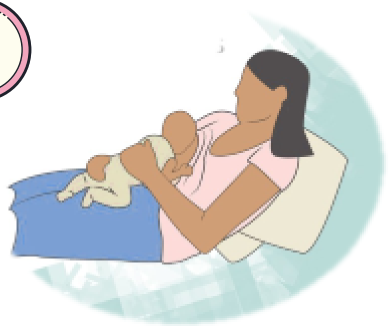

Imagens baseadas nas recomendações do Ministério da Saúde.

POSIÇÕES PARA AMAMENTAR GÊMEOS

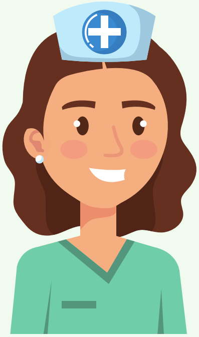

É importante lembrar que uma das causas do nascimento prematuro é a gestação de gêmeos. Por isso ensinaremos abaixo algumas posições que ajudem a mãe a amamentar nesses casos!

1 lateral
1 tradicional

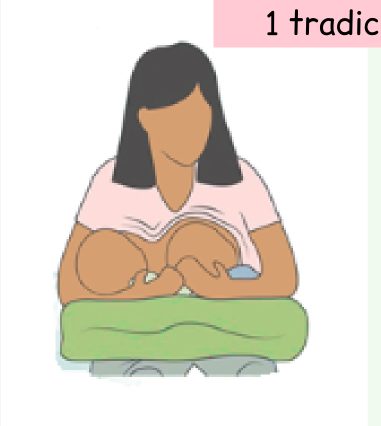

De frente (tipo cavalinho)

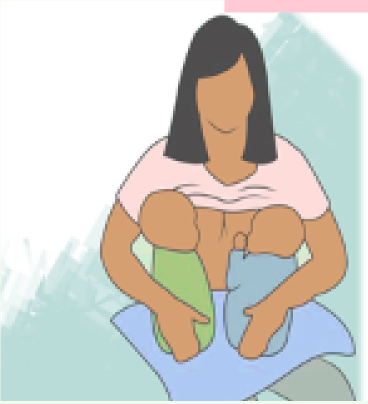

Cruzados

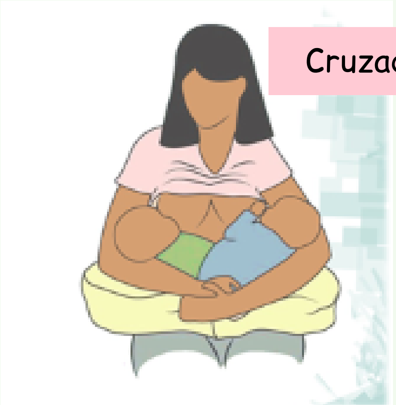

Lateral (tipo bola de futebol americano)

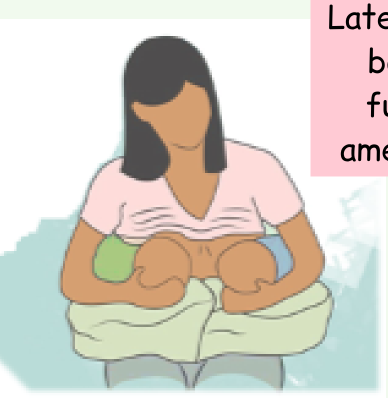

Imagens baseadas nas recomendações do Ministério da Saúde.

POSIÇÃO PÓS AMAMENTAÇÃO

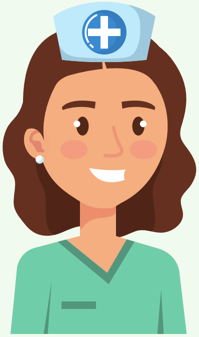

Também é importante lembrar de posicionar o bebê para arrotar após a mamada! Essa simples ação evita o refluxo (algo muito comum entre os recém-nascidos)!

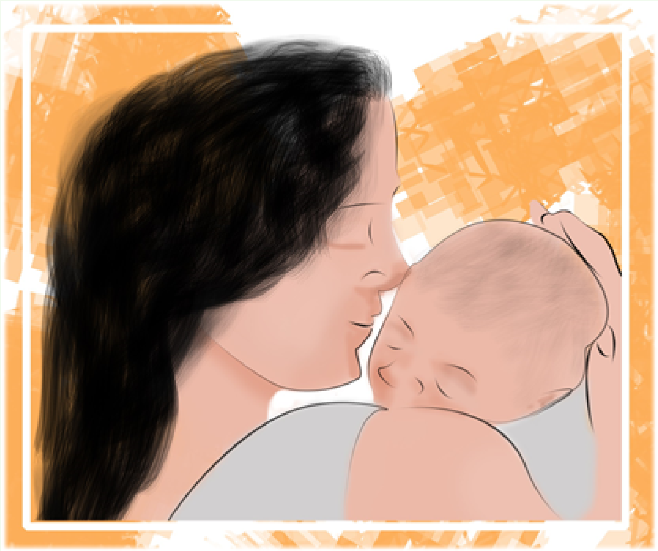

- A melhor posição para colocar o bebê para arrotar é semelhante a posição canguru: o bebê deve estar em pé e sua cabecinha apoiada nos ombros do cuidador.

CURIOSIDADE

Mas então, o que é o Refluxo?!

- O refluxo ocorre quando há a volta involuntária do alimento que está no estômago para o esôfago.

- Esse retorno do alimento causa principalmente sensação de queimação e também náuseas e vômitos. E pode estar presente no bebê prematuro devido imaturidade muscular (esfincter).

- O esfincter esofágico (músculo circular) funciona como uma porta que abre e fecha após a passagem do alimento, como ainda há uma certa imaturidade presente no prematuro, nem sempre após a passagem do alimento ele se fecha corretamente, podendo gerar o refluxo e seus sintomas.

- Aqui estão listado alguns sinais e sintomas que necessitam de avaliação do profissional de saúde, por isso, é importante sempre estar atento!

- Vômito várias vezes ao dia após mamar, em grande quantidade;
- Dor ou cólica às vezes sinalizadas por choro, engasgos e tosse;
- Perda de peso;
- Comprometimento do desenvolvimento;
- Chiado, dificuldade para respirar e otites (inflamação dos ouvidos).

RETIRANDO E ARMAZENANDO O LEITE MATERNO

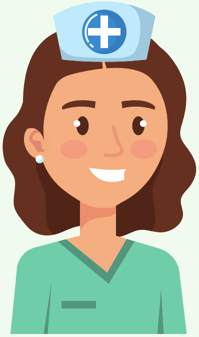

Então vamos aprender a realizar retirada e o armazenamento do seu leite de maneira simples e prática?

INFORMAÇÕES IMPORTANTES

- Devido à prematuridade, o bebê poderá mamar pouco, por isso, pode ser necessário acordá-lo para ofertar o leite materno retirado;

- Em casos de ausência materna, é preciso retirar o leite e armazenar para oferta-lo depois;

- É importante lembrar também que caso o bebê tenha dificuldade de sugar, o leite retirado deve ser ofertado no copinho, para que o bebê não faça confusão de bico e acabe se acostumando com o a mamadeira e deixando o peito.

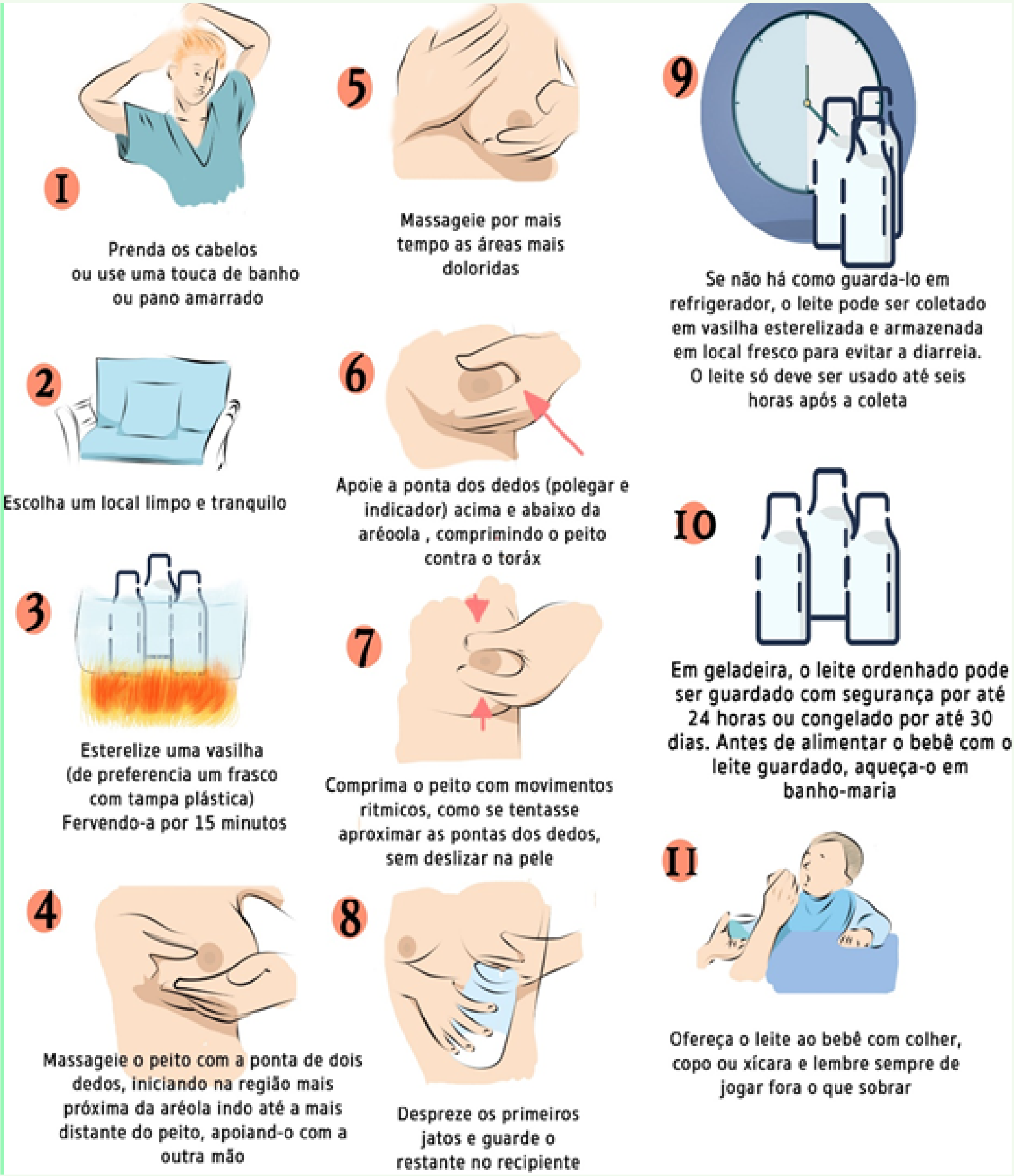

Recomendações do Ministério da Saúde.

VAMOS ACABAR COM ALGUNS MITOS RELACIONADOS À AMAMENTAÇÃO DO BEBÊ PREMATURO?!

"O bebê pode ficar mal acostumado se não tiver horários para mamar!"

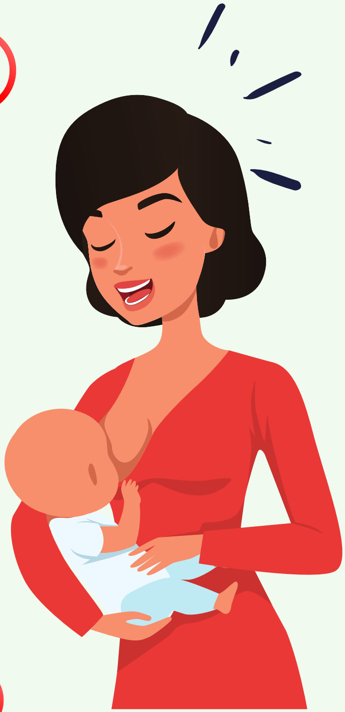

"Seu leite é fraco e não mata a fome do bebê, por isso ele chora!"

"O bebê é muito pequeno e sensível, não tem força para sugar no peito!"

"Não faz diferença na vida do bebê prematuro se ele mamar ou não!"

- É bem provável que em algum momento da vida você já tenha ouvido alguma dessas afirmações. Por isso que agora vamos te dizer se realmente é verdade ou não!

"O bebê pode ficar mal acostumado se não tiver horários para mamar!"

MITO

NA VERDADE...

- O que ocorre, é que bebê prematuro é um pouco mais lento para sugar, e se cansa mais rapidamente, por isso, as vezes é necessário que se faça uma pausa para ele respirar e depois retorna naturalmente a mamada!

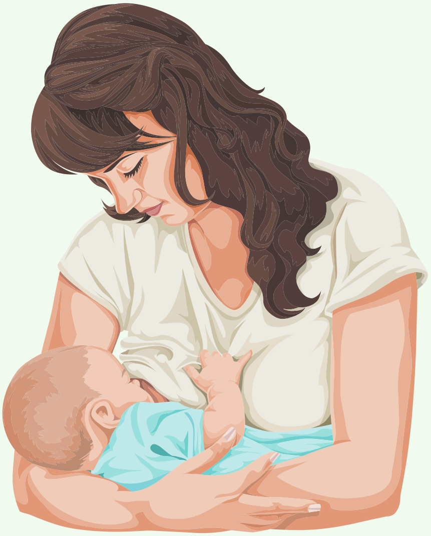

"O bebê é muito pequeno e sensível, não tem força para sugar no peito!"

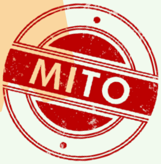

LIVRE DEMANDA!

- O bebê deve ser amamentado a hora que ele quiser e quando ele quiser, principalmente o bebê prematuro. E por que isso ?!;

- Porque o bebê prematuro tem necessidade calórica aumentada. Dessa forma, caso o bebê seja muito sonolento é necessário que o acorde para que ele possa mamar;

- À medida que crescem, os bebês vão se acomodando a um ritmo próprio de frequência e duração da mamada.

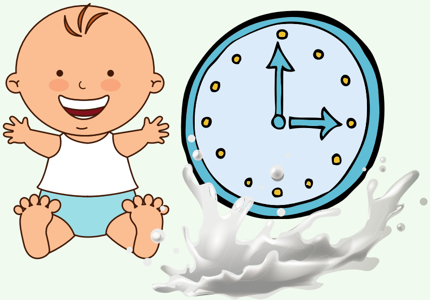

"Não faz diferença na vida do bebê prematuro se ele mamar ou não!"

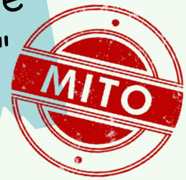

BENEFÍCIOS DA AMAMENTAÇÃO PARA O BEBÊ PREMATURO

- Na amamentação, o bebê recebe os anticorpos da mãe para proteção contra diarréia e diversos tipos de infecções, principalmente as respiratórias;
- O leite materno diminui as chances do bebê desenvolver alergias, colesterol alto, diabetes e obesidade;
- O aleitamento materno também ajuda a criança a desenvolver-se bem, fisicamente e emocionalmente;
- É um excelente exercício para o desenvolvimento da face e da fala;
- É importante para que a criança tenha dentes fortes e alinhados, e também para que o bebê tenha uma boa respiração.

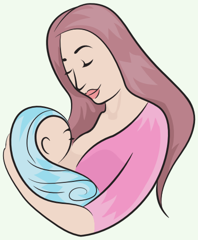

"Seu leite é fraco e não mata a fome do bebê, por isso ele chora!"

MITO

NA VERDADE...

- Quando a mãe está em estado mais relaxado, ocorre, picos de ocitocina (o hormônio que auxilia na liberação do leite materno), que é quando o leite fica rico em gordura, independente se no início, meio ou no fim da mamada, sustentando mais o bebê;

- Outra dica super importante é esvaziar a primeira mama antes de passar para a outra. E na próxima mamada, iniciar pela última.

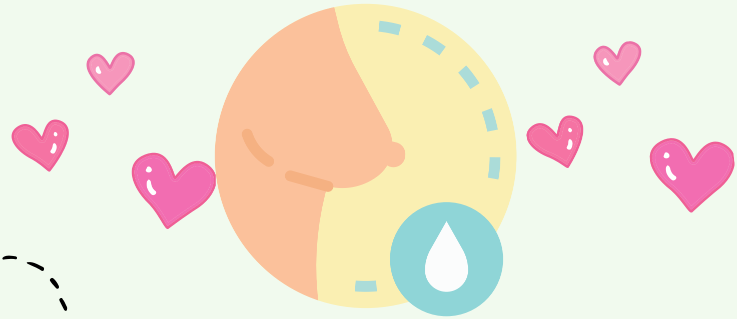

HORA DO BANHO!

- A hora do banho é um momento bem importante para o bebê prematuro, porque além de deixá-lo limpinho, é um momento propício para estreitar os laços afetivos através de conversas, olhares, carinho e muito amor!

- Esse momento pode gerar insegurança para os cuidadores, por achar que o bebê é frágil, pequeno e sensível. Mas é possível vivenciar esse momento da melhor maneira possível!

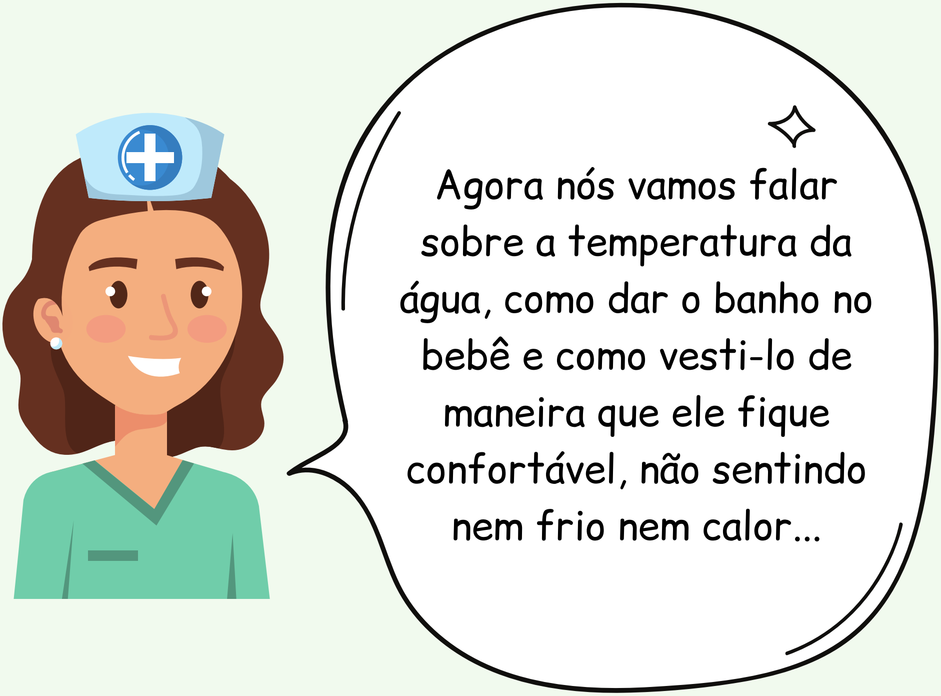

PASSOS PARA AJUDAR NO BANHO DO SEU BEBÊ!

1 O primeiro passo para o banho é a separação do material que você irá utilizar:

Fralda

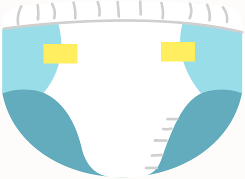

Toalha

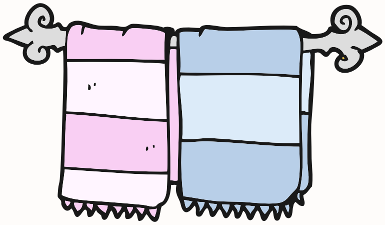

Sabonete neutro

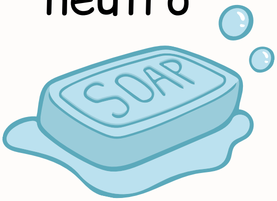

Shampoo

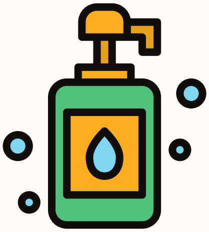

Contonete

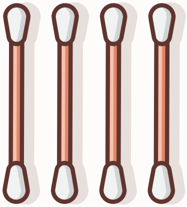

Álcool 70°

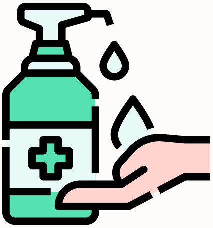

Água morna

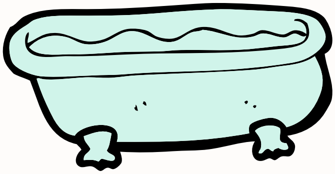

2 O segundo passo para o banho é a conferência da temperatura da água:

- A temperatura adequada da água é entre 36,5° e 37°C e pode ser medida com termômetro.
- Caso a família não tenha termômetro, basta colocar a região interna no braço na água (região do pulso), se estiver agradável para você, também estará para o bebê.
- O local para o banho deve estar fechado e sem a circulação de vento (fechar todas as janelas e portas).

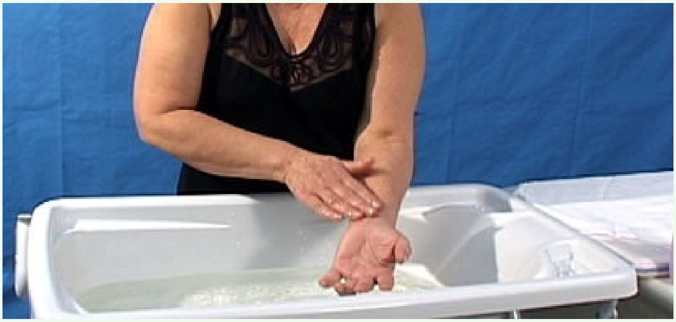

Imagens baseadas nas recomendações do Ministério da Saúde.

3

O banho do bebê prematuro não tem uma ordem específica a ser seguida, mas aqui tem um exemplo que pode ajudar nesse momento tão especial na vida do bebê:

A) Pode-se iniciar o banho limpando os olhos do bebê apenas com água.

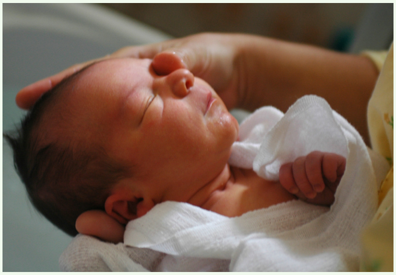

B) Em seguida lava-se e seca-se a cabeça do bebê, com ele ainda enrolado na toalha.

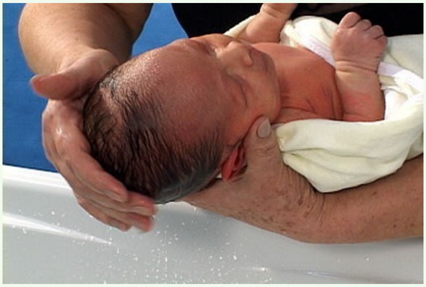

C) Logo depois desenrola-o da toalha e o imerge na banheira, lavando primeiro a parte da frente do corpinho

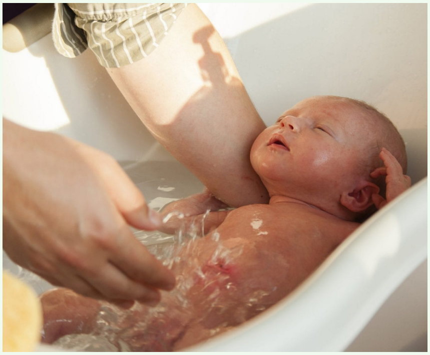

Imagens baseadas nas recomendações do Ministério da Saúde.

D) Após lavar a parte da frente, vire-o, para lavar a parte de trás do corpinho e genetália.

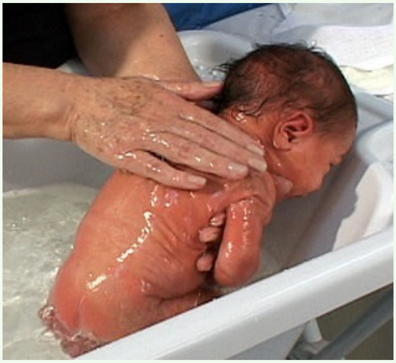

Imagens baseadas nas recomendações do Ministério da Saúde.

DICAS IMPORTANTES PARA AJUDAR NO BANHO DO SEU BEBÊ!

- Higienize a banheira com água e sabão ou álcool antes e depois do banho. A água deve ocupar cerca de 5 cm do volume da banheira.
- Nos primeiros meses, o shampoo é dispensável, já que os bebês costumam ter pouco cabelo. Mas, depois opte por escolher um produto de preferência sem perfume.
- Prefira banhar o bebê no horário mais quente do dia, ou seja, perto da hora do almoço. Se o único horário disponível for a noite, evite sair após o banho.
- Feche as janelas e portas para evitar correntes de ar e deixe tudo o que for usar em local de fácil acesso. E procure um local familiar para dar banho no bebê, pode ser no seu quarto ou no banheiro mesmo!
- E lembre-se: a confiança e a segurança virão com o tempo!

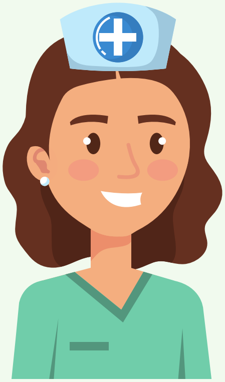

Agora vamos falar sobre as roupinhas do bebê! O bebê prematuro é mais sensível e necessita de um cuidado maior na hora de vesti-lo, para que não perca calor e nem peso. Por isso, uma boa dica é vesti-lo de acordo com o clima da sua região.

- Abaixo de 16°C

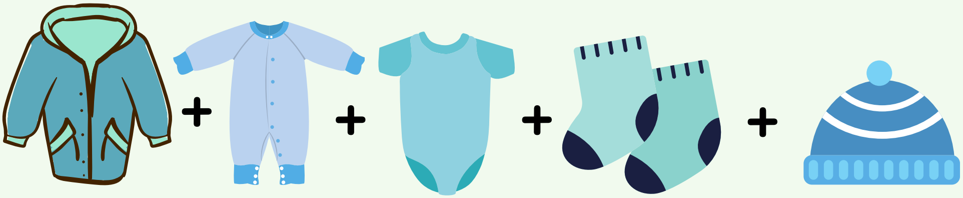

- Entre 16° e 17°C

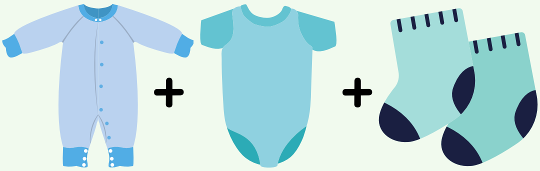

- Entre 18° e 21°C

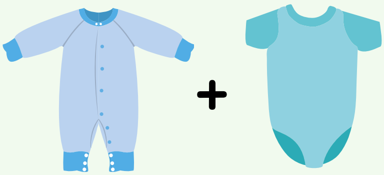

- Entre 22° e 23°C

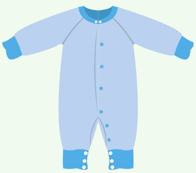

- Entre 24° e 25°C

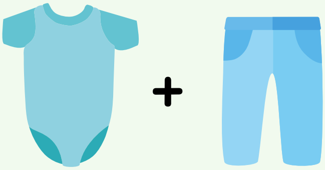

- Acima de 26°C

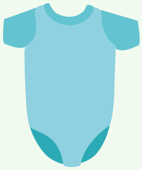

INFORMAÇÕES IMPORTANTES QUANTO À SAÚDE DO SEU BEBÊ!

- Os bebês prematuros possuem a capacidade de perder calor com mais facilidade, devido à sua área corporal ser menor (menos tecido cutâneo);

- Dessa forma, é importante ficar de olho a quantidade de vestimentas de acordo com a temperatura do local, pois, excesso de vestimentas pode fazer com que ele perca calor e consequentemente peso corporal.

- O bebê prematuro também se resfria com muita facilidade, e esse excesso de resfriamento pode gerar no bebê alguns problemas como:

- Dificuldade de respirar;
- Problemas graves nos rins;
- Dificuldade de ganhar peso;
- Inflamação no intestino;
- Sangramentos no coração e outros.

- Por isso, é importante estar sempre atento à temperatura do ambiente e ao tipo de roupinha utilizada no bebê!

ESTREITANDO OS LAÇOS AFETIVOS

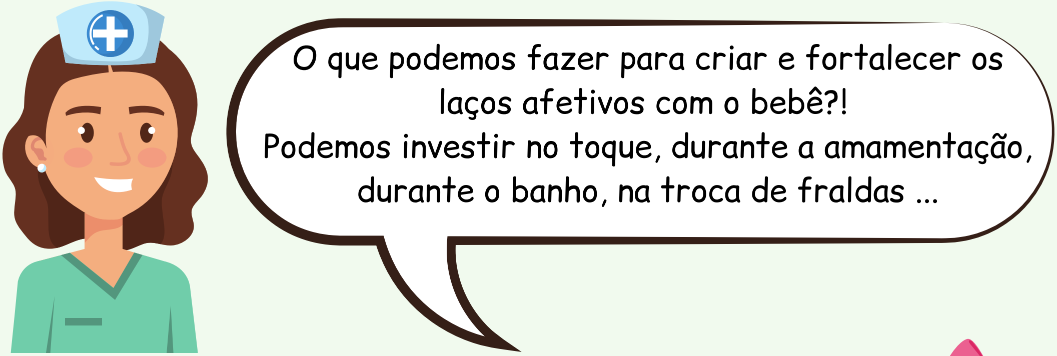

- O que podemos fazer então para promover esses laços?!

- Conversar com o seu bebê, durante os cuidados diários, como no banho e durante a amamentação;

- Um olhar prolongado para o seu bebê, sorrir, acariciar, aconchegar, o abraçar e o beijar, são comportamentos que certificam a existência desse afeto.

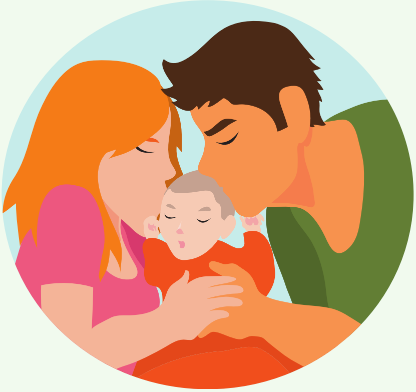

- E então, quais os benefícios de construir e estreitar os laços afetivos? Vamos lá...

- Gera o conhecimento do seu próprio filho, sendo capaz de identificar suas necessidades e cuidar deles de acordo as suas particularidades;

- Possui ligações diretas com o crescimento e desenvolvimento do bebê prematuro, seja ele emocional, social e intelectual;

- Melhora a estabilidade fisiológica:

- Diminui o tempo de internação hospitalar;
- Melhora o estado de humor da mãe;
- Auxilia no aleitamento materno e outros.

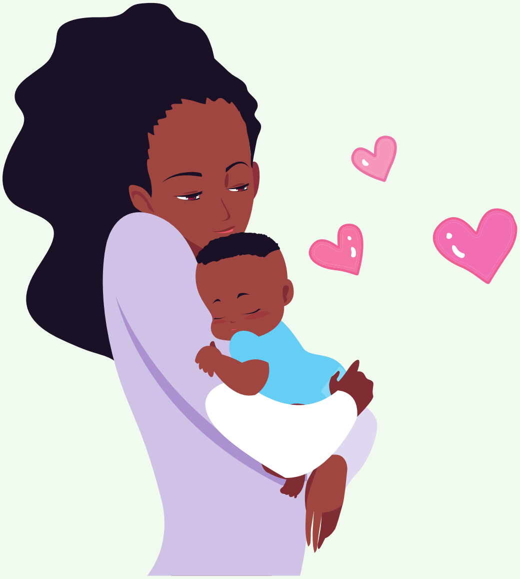

CUIDAR DO AMBIENTE
TAMBÉM É CUIDAR DA SAÚDE DO BEBÊ!

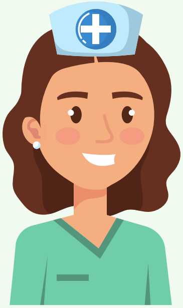

Agora vamos falar agora sobre pequenas ações que vão ajudar na saúde do bebê, sem que ele fique privado de tudo!

- Cuidado ao receber visitas. Limitar o número de pessoas a 1 por vez;

- Manter sempre a casa arejada e limpa, procurar limpar com produtos neutros e não deixar mofo nem umidade;

- Lavagem das mãos antes de pegar, cuidar e alimentar o bebê;

- Evitar ambiente com muitas pessoas;

- Manter as vacinas todas em dia.

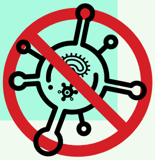

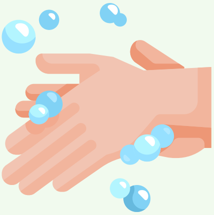

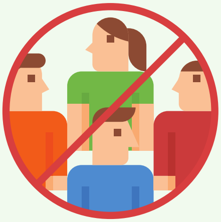

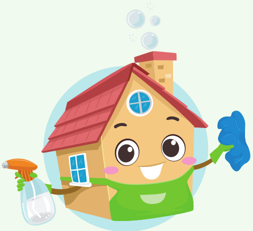

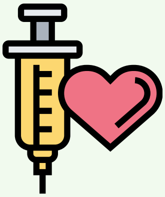

TODO APOIO É BEM VINDO!

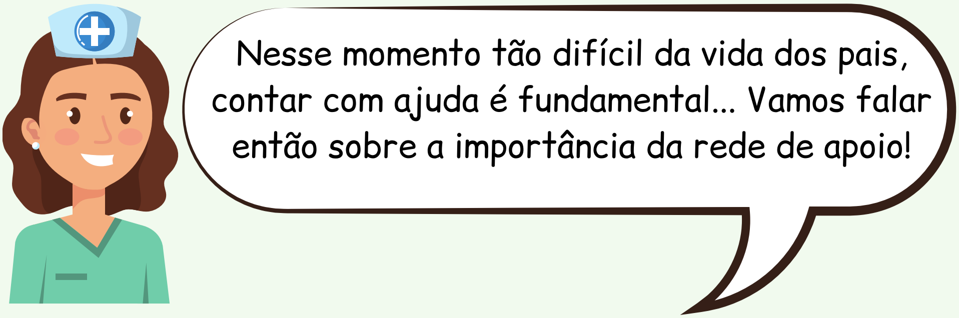

- Rede de apoio são pessoas próximas que podem ajudar a cuidar do bebê, seja ela familiar ou de amigos.

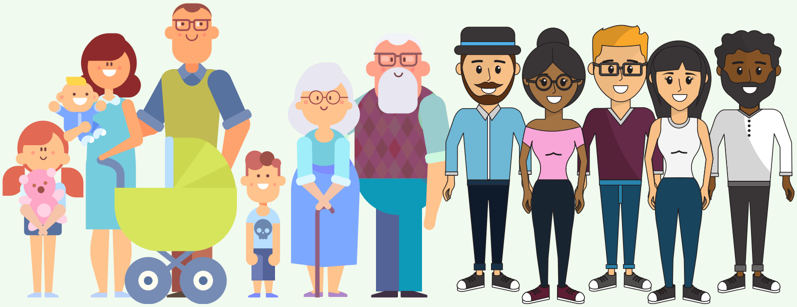

- Você sabia que a rede de apoio tem muitos benefícios para o bebê prematuro e para a família durante esse momento?

- Ajuda no equilíbrio das emoções, diminuindo o medo e a ansiedade dos cuidadores;
- Aumenta a autoconfiança na hora de realizar os cuidados domiciliares e assim ajuda e diminuir a insegurança;
- É um grande aliado para adaptação dos cuidadores diante da nova realidade e ajuda da dinâmica familiar.

VOCÊ SABIA?

1

Que não é aconselhado utilizar mamadeira, chupeta ou qualquer outro tipo de bico de silicone, pois pode gerar a "confusão de bico"?!

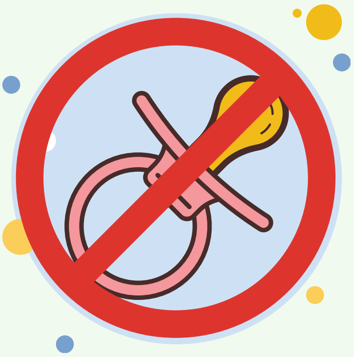

- A confusão de bico é causada quando o bebê se acostuma com o bico artificial pois esse é ofertado a ele várias vezes.

- Isso ocorre pois o bico artificial possui: pega, forma de sugar, consistência, textura e elasticidades diferentes do bico do peito materno.

- Logo, o bebê acostuma-se com o bico artificial e acaba rejeitando o bico do peito materno, gerando assim o abandono da amamentação!

VOCÊ SABIA?

2

Que existe uma forma de oferecer o leite materno ao bebê prematuro sem precisar utilizar bicos artificiais?!

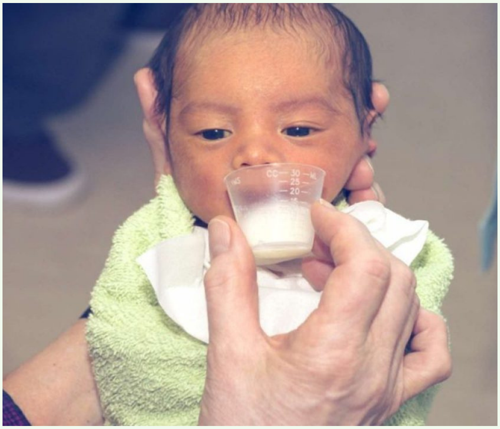

Imagens baseadas nas recomendações do Ministério da Saúde.

- Pode-se oferecer o leite materno no copinho, essa ferramenta simples e prática ajuda a estimular a ingestão do leite materno e evita a confusão de bico!

VOCÊ SABIA?

3

Que é necessário alguns cuidados para quando ofertar o leite materno no copinho?

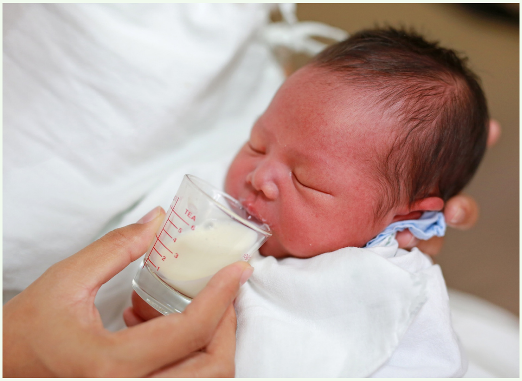

Imagens baseadas nas recomendações do Ministério da Saúde.

- Primeiramente é importante realizar a lavagem das mãos;
- Em seguida posicionar o bebê na posição semissentada;
- Conferir a temperatura do leite, que deve estar morno (lembrar de esquentá-lo em banho maria e não deixar ferver);
- Para ofertar o leite, pode-se posicionar a borda do copinho em seu lábio superior, dessa forma o bebê não empurra o copinho;
- E, com paciência esperar que o bebê sugue e engula o leite, sem precisar força-lo.

VOCÊ SABIA?

4

Que mamar no peito é essencial para os bebês pois, trabalha toda a musculatura facial. Pelo contrário, na mamadeira, a sucção realizada não usa de maneira correta os músculos faciais, podendo gerar no bebê problemas dentários e na fala.

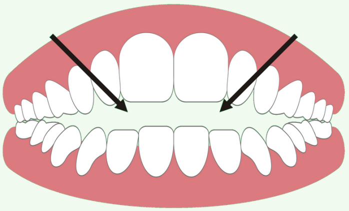

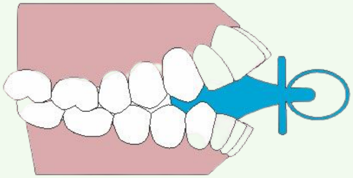

- Algumas consequências do uso de bicos artificiais são:

- Altera a posição dos dentes, podendo deixar a mordida aberta ou a mordida cruzada;

- A musculatura dos lábios e da língua podem ficar flácidos, o que pode dificultar na sucção no peito, na mastigação e na fala;

- Interfere na respiração;

- Há crianças que deixam de aceitar o peito, já que o bico artificial é diferente.

Imagens baseadas nas recomendações do Ministério da Saúde.

VOCÊ SABIA?

5

Que cerca de 30% das gestações de gêmeos entram em trabalho de parto prematuro?!

- Gêmeos, trigêmeos ou mais bebês tendem a nascer antes do tempo;

- Os fatores que levam a isso têm pouca ligação com seu comportamento ou estilo de vida da mãe, alimentação e prática de exercícios físicos;

- Uma alimentação balanceada durante a gravidez também é fundamental, assim os bebês terão mais chances de nascer saudáveis e com um bom peso;

- Uma dica importante é procurar fazer pelo menos cinco refeições por dia (três refeições e dois lanchinhos nutritivos), mesmo que não esteja com muita fome.

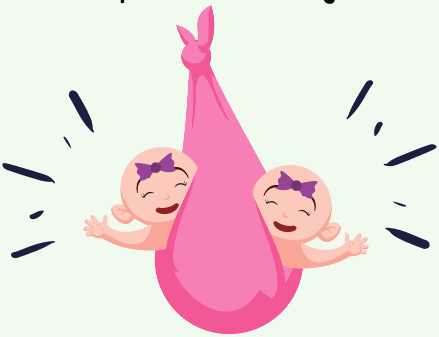

VOCÊ SABIA?

6

Que os recém-nascidos prematuros emitem sinais de alerta, e quando emitidos é preciso buscar ajuda no pronto atendimento mais próximo?!

- Alguns sinais de alerta são:

- Excesso de sonolência e baixa resposta aos estímulos;
- Xixi de coloração escura e poucas vezes ao dia;
- Pele roxinha ou muito pálida;
- Convulsões;
- Vômitos frequentes;
- Apresentar extremos de temperatura: febre (acima de 37,5°c) ou hipotermia (menos de 36°c);
- Barriguinha aumentada e endurecida;

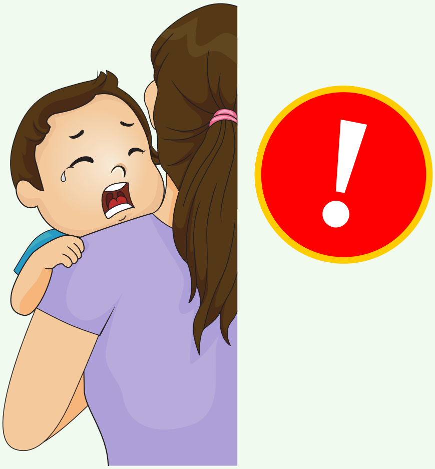

INFORMAÇÃO IMPORTANTE!

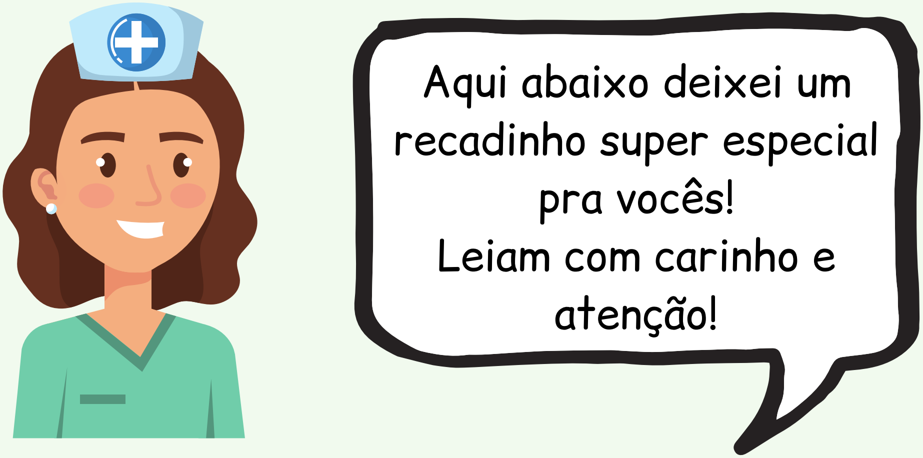

Queridos familiares!

Eu, Enfermeira Catarina, estou muito feliz em poder participar dessa jornada de aprendizado com vocês, e poder contribuir com informações que os ajudarão no cuidado com o bebê prematuro.

Mas, preciso lembrá-los que essa cartilha não contém todas as respostas para as questões que surgirão no dia a dia, por isso, é de extrema importância comparecer às consultas de puericultura, para que dessa forma, o seu bebê se desenvolva da melhor maneira possível!

Com carinho, Enfa. Catarina

REFERÊNCIAS

AGOSTINHO, C; FRIAS, A. Ocitocina, uma hormona amiga da amamentação. 2018.

ALMEIDA, L.I.V; RAMOS, S.B; FIGUEIREDO, G.LA. Apoio e rede social no contexto urbano: percepções de mães de crianças prematuras. Aletheia, v. 52, n. 1, 2019.

BOTÊLHO, S.M et al. O cuidar materno diante do filho prematuro: um estudo das representações sociais. Revista da Escola de Enfermagem da USP, v. 46, n. 4, p. 929-934, 2012.

DA SILVA, J.M.F. Prematuridade na gravidez gemelar. 2018.

FONSECA, L.M.M; SCOCHI, C.G.S. Cuidados com o bebê prematuro: orientações para a família. FIERP/EERP-USP, 2009. Disponível em: <http://bvsms.saude.gov.br/bvs/publicacoes/cuidados_bebe_prematuro_3ed.pdf>

LIMA, R.O et al. Intervenção de enfermagem-primeiro banho do recém-nascido: estudo randomizado sobre o comportamento neonatal. Acta Paulista de Enfermagem, v. 33, 2020.

MEDEIROS, C.C. Conhecimento e cuidado parental na promoção do desenvolvimento da criança nascida prematura. 2018.

MÉIO, M.D.B.B et al. Amamentação em lactentes nascidos pré-termo após alta hospitalar: acompanhamento durante o primeiro ano de vida. Ciência & Saúde Coletiva, v. 23, p. 2403-2412, 2018.

MOITA, C.E et al. O enfermeiro no acolhimento aos pais de recém-nascido prematuro na unidade de terapia intensiva neonatal. Revista de Trabalhos Acadêmicos-universo Salvador, v. 1, n. 6, 2018.

NASCIMENTO, A.C.S.T et al. Redes Sociais de Apoio as famílias de prematuros que vivenciam a hospitalização: Um estudo Transcultural. Revista Eletrônica Acervo Saúde, n. 37, p. e1986-e1986, 2019.

NUNES, R.C.T et al. Família e recém-nascido: diretrizes para uma nova prática de enfermagem. Enfermería: Cuidados Humanizados, v. 5, n. 2, p. 19-24, 2016.

ROCHA, G.M.N et al. Dúvidas maternas na alta hospitalar do recém-nascido. Revista Univap, v. 25, n. 49, p. 93-103, 2019.

SALES, I.M.M et al. Contribuições da equipe enfermagem na segunda etapa do Método Canguru: Implicações para a alta hospitalar do recém-nascido. Escola Anna Nery, v. 22, n. 4, 2018.

SBP, Sociedade Brasileira de Pediatria.Guia de Fotoproteção na Criança e Adolescente.1ª edição.Rio de Janeiro.2017.

SILVA, E.B; SILVA, M.S. Dificuldades enfrentadas pelas mães na amamentação do recém-nascido pré-termo em UTI's neonatal. 2019.

SILVA, F.B et al. Complicações materno-fetais de gestações gemelares. Cadernos da Medicina-UNIFESO, v. 2, n. 1, 2019.

VILAR, R.L.C et al. Cartilha cuidados com o bebê prematuro: orientações para a família: uma estratégia de educação em saúde na atenção básica. 2017.

Universidade Federal do Espírito Santos
Centro Universitário Norte do Espírito Santo
Departamento de Ciências da Saúde
Núcleo de Pesquisa em Saúde

ISBN: 978-65-00-17168-6

9 786500 171686
